# Bacterial periplasmic sialic acid-binding proteins exhibit a conserved binding site

**DOI:** 10.1107/S139900471400830X

**Published:** 2014-06-24

**Authors:** Thanuja Gangi Setty, Christine Cho, Sowmya Govindappa, Michael A. Apicella, S. Ramaswamy

**Affiliations:** a Institute for Stem Cell Biology and Regenerative Medicine, NCBS Campus, GKVK Post, Bangalore, Karnataka 560 065, India; b Department of Microbiology, Carver College of Medicine, University of Iowa, Iowa City, IA 52242-1109, USA

**Keywords:** sialic acid-binding proteins, structure binding, molecular mimicry

## Abstract

Sialic acids are a family of related nine-carbon sugar acids that play important roles in both eukaryotes and prokaryotes. These sialic acids are incorporated/decorated onto lipooligosaccharides as terminal sugars in multiple bacteria to evade the host immune system. Many pathogenic bacteria scavenge sialic acids from their host and use them for molecular mimicry. The first step of this process is the transport of sialic acid to the cytoplasm, which often takes place using a tripartite ATP-independent transport system consisting of a periplasmic binding protein and a membrane transporter. In this paper, the structural characterization of periplasmic binding proteins from the pathogenic bacteria *Fusobacterium nucleatum*, *Pasteurella multocida* and *Vibrio cholerae* and their thermodynamic characterization are reported. The binding affinities of several mutations in the Neu5Ac binding site of the *Haemophilus influenzae* protein are also reported. The structure and the thermodynamics of the binding of sugars suggest that all of these proteins have a very well conserved binding pocket and similar binding affinities. A significant conformational change occurs when these proteins bind the sugar. While the C1 carboxylate has been identified as the primary binding site, a second conserved hydrogen-bonding network is involved in the initiation and stabilization of the conformational states.

## Abstract 2

Structure–function studies of sialic acid-binding proteins from *F. nucleatum*, *P. multocida*, *V. cholerae* and *H. influenzae* reveal a conserved network of hydrogen bonds involved in conformational change on ligand binding.

## 1. Introduction   

Bacteria and their animal hosts are constantly co-evolving, with the former using many strategies to avoid the immune defences of the latter. Many Gram-negative bacteria mask their alien identity by decorating their outer layer with various forms of a linear monosaccharide that is also found on the surface of most animal cells: sialic acid (SA). By adding this nine-carbon sugar acid to the outermost end of certain glycolipids that coat the bacterial surface, the pathogen can primarily derail the activation of the host’s most primitive immune response: the complement system (Hood *et al.*, 1999 ▶; Harvey *et al.*, 2001 ▶; Severi *et al.*, 2007 ▶; Johnston *et al.*, 2008 ▶; Freire-de-Lima *et al.*, 2012 ▶).

While eukaryotic cells can produce SA *de novo*, this ability is not common in bacteria, and most SA-decorating bacteria identified to date scavenge it from their host. The most common means of procuring exogenous SA is a multi-stage operation that at the least involves (i) capturing an extracellular sialic acid molecule *via* a specialized receptor and transporting it through the plasma membrane into the cell, (ii) linking the SA to a glycolipid (lipooligosaccharide or lipo­polysaccharide) and finally (iii) embedding the newly formed SA–glycolipid complex within the plasma membrane with the SA facing outwards. One of the more recently reported mechanisms for the capture and transport of SA involves a type of transporter used in many bacteria for the uptake of a range of organic acid ligands: the tripartite ATP-independent periplasmic (TRAP) transporter (Fischer *et al.*, 2010 ▶). The first SA-decorating bacterium to use the TRAP transporter was recently identified in a non-encapsulated strain of the human pathogen *Haemophilus influenzae* (Kelly & Thomas, 2001 ▶; Severi *et al.*, 2005 ▶). Evidence indicates that other human pathogens, *Fusobacterium nucleatum* and *Vibrio cholerae*, and the animal pathogen *Pasteurella multocida* also use TRAP transporters for SA uptake (Kapatral *et al.*, 2002 ▶; Steenbergen *et al.*, 2005 ▶; Almagro-Moreno & Boyd, 2009 ▶; Chowdhury *et al.*, 2012 ▶; Mulligan *et al.*, 2012 ▶). In three of these four species the role of SA utilization in virulence has also been strongly supported. For *V. cholerae*, its normal virulence in gastric conditions is more related to the cholera toxin (Almagro-Moreno & Boyd, 2009 ▶).

The possibility of therapeutically targeting a large number of bacterial pathogens *via* the SA-TRAP transporter underlines the importance of a thorough understanding of its structure and function. To date, only one SA-TRAP transporter binding protein has been studied structurally: that from *H. influenzae*. This transporter consists of an integral membrane component (SiaT), which is a membrane transporter, and an extracellular solute receptor (SiaP), which binds the main mammalian form of SA (Neu5Ac; Mulligan *et al.*, 2009 ▶). These soluble proteins belong to the Pfam family of bacterial extracellular solute-binding proteins (family 7). These proteins bind to a variety of solutes that have a conserved carboxylate-type group. They all have a size that varies between 300 and 310 amino acids. While we expect the overall structures of all of these proteins to be similar (Fischer *et al.*, 2010 ▶), the sequence identity between the two most divergent proteins reported in this work is 47%. The details of their molecular interactions are thus different and it is important to determine the structures of the individual proteins in order to dissect the conserved molecular details for functional analysis.

Relatively little is known about the structure of the integral membrane component(s) of any TRAP transporter. In contrast, the extracellular solute receptors, and SiaP from *H. influenzae* in particular, have attracted considerable attention. SiaP is a protein with two domains that fold around a well defined pocket. When SA binds to SiaP, the pocket closes over it as the two domains bend at a hinge region (Müller *et al.*, 2006 ▶; Johnston *et al.*, 2008 ▶). The binding itself is initiated when atoms of the carboxylate group of Neu5Ac interact with residues Arg147 and Arg127 of domain II of SiaP, forming salt bridges. Structural, mutagenesis and complementation studies have also indicated that other Neu5Ac atoms and SiaP residues are involved in orienting Neu5Ac within the pocket, in domain folding and in linking Neu5Ac to residues of domain I of SiaP. These findings concur with structural studies of the extracellular solute receptors of several bacterial species that scavenge other extracellular solutes (Fischer *et al.*, 2010 ▶). This emerging picture of a conserved mechanism across a diverse range of pathogens is a strong motivator to compare and contrast the structure of SiaP from *H. influenzae* (Hi-SiaP) with its analogues in other Gram-negative bacteria that use the SA-TRAP transporter.

In this study, we characterize the binding site of the extracytoplasmic solute receptors of *Fusobacterium nucleatum* (Fn), *Pasteurella multocida* (Pm) and *Vibrio cholerae* (Vc) by resolving the crystal structures of Fn-SiaP, Pm-SiaP (Neu5Ac-bound) and Vc-SiaP (unbound). In addition to this, we also used isothermal calorimetry to (i) investigate the ligand specificities of Fn-SiaP, Pm-SiaP and Vc-SiaP by determining their binding affinities for Neu5Ac and the nonhuman variant Neu5Gc and (ii) further explore the ligand-binding roles of various residues in the Hi-SiaP pocket by measuring the binding affinities (for Neu5Ac) of eight Hi-SiaP mutants used in a prior complementation study. Finally, we also examined whether *V. cholerae* could incorporate Neu5Ac into its surface glycolipids, since this has not been demonstrated before.

## 2. Experimental procedures   

### 2.1. Protein expression and purification (Hi-SiaP, Fn-SiaP, Pm-SiaP and Vc-SiaP)   

Wild-type Hi-SiaP and its E67A, E186Q, N187Q, R127K, R127A, T64K, T64R and H209A mutants were expressed and purified by a modification of the method of Johnston *et al.* (2008 ▶).

Genes corresponding to the periplasmic binding proteins from *F. nucleatum* (Fn-SiaP; NCBI Reference Sequence NP_604366.1) and *P. multocida* (Pm-SiaP; NCBI Reference Sequence NP_246648.1) were synthesized by GenScript in pET-28a (Novagen) with a C-terminal His tag at the *Nco*I and *Xho*I sites without signal peptide. The DNA encoding *V. cholerae* (Vc-SiaP; NCBI Reference Sequence NP_231414.1) was a gift from Dr Linda McCarter, Department of Microbiology, The University of Iowa, USA. The corresponding gene for Vc-SiaP was PCR-amplified using the forward primer 5′-GCC GGA ATT CGC GAC GAC TTT AAAG-3′ and the reverse primer 5′-CCG CTC GAG CAT TGC TGC-3′. The PCR product was cloned into pET-21a (Novagen) vector containing a C-terminal His tag at the *Eco*RI and *Xho*I sites. Positive clones were confirmed by restriction digestion and then by sequencing.

The recombinant plasmids were expressed in *Escherichia coli* BL21 (DE3) cells for protein expression. The cells were grown in LB medium containing either kanamycin (for Fn-SiaP and Pm-SiaP) or ampicillin (for Vc-SiaP) at 37°C to an OD_600_ of 0.6. The cells were then induced with 100 µ*M* IPTG. After induction, the cells were allowed to grow at 25°C for 4 h. The cells were harvested and centrifuged at 13 000 rev min^−1^ for 30 min and each 1 l cell pellet was resuspended in 25 ml resuspension buffer (20 m*M* HEPES, 150 m*M* NaCl, 5 m*M* imidazole pH 8.0) with a protease-inhibitor cocktail without EDTA (Roche). Lysozyme and DNase were added to each 1 l culture pellet and the cells were lysed using an EmulsiFlex at 103 MPa. The lysate was centrifuged at 13 000 rev min^−1^ for 30 min.

#### 2.1.1. Purification of Fn-SiaP and Pm-SiaP   

A Ni-affinity column (Bio-Rad) was used as the first purification step for Fn-SiaP and Pm-SiaP using a Profinia system (Bio-Rad). After loading the sample onto the column, the column was first washed with resuspension buffer and then with ten column volumes (CV) of 20 m*M* HEPES, 500 m*M* NaCl, 5 m*M* imidazole pH 8.0. The protein was then eluted with 4 CV of 20 m*M* HEPES, 150 m*M* NaCl, 500 m*M* imidazole pH 8.0. The eluate was dialyzed overnight into 20 m*M* HEPES pH 7.5, 10 m*M* NaCl. The Pm-SiaP protein was further purified using anion-exchange chromatography (HiTrap Q FF, GE Healthcare). After the protein had been loaded onto the column, it was washed with 20 m*M* HEPES pH 7.5, 10 m*M* NaCl. The protein was then eluted by a gradient to 1 *M* NaCl in the same buffer. The protein eluted at a salt concentration of 150 m*M*. Following Ni-affinity purification, the Fn-SiaP protein was further purified by cation-exchange chromatography (HiTrap SP FF, GE Healthcare). Nonspecifically bound proteins were washed out with 20 m*M* HEPES pH 7.5, 10 m*M* NaCl, and Fn-SiaP was eluted by a gradient to 1 *M* NaCl in the same buffer. The protein eluted at a salt concentration of 150 m*M*. The fractions containing the proteins were pooled for further purification using size-exclusion chromatography (Superdex 75, GE Healthcare) with equilibration in 20 m*M* HEPES pH 8.0, 10 m*M* NaCl. In this step, the proteins were purified together with Neu5Ac (for crystallization) or without Neu5Ac (for ITC experiments).

#### 2.1.2. Purification of Vc-SiaP   

The Vc-SiaP lysate was mixed with BD TALON metal-affinity resin (BD Biosciences). The mixture was incubated at 4°C for 16 h. This mixture was then centrifuged at 1000 rev min^−1^ for 5 min to pellet the beads. The supernatant was removed and the beads were washed four times with 10 CV resuspension buffer using a 50 ml disposable vacuum Steriflip 0.22 µm filtration system (Millipore). The protein was then eluted with 6 CV elution buffer (20 m*M* HEPES, 150 m*M* NaCl, 500 m*M* imidazole pH 8.0). The eluate was dialyzed overnight into a buffer consisting of 20 m*M* HEPES pH 8.0, 10 m*M* NaCl to remove imidazole. The dialyzed protein was concentrated using an Amicon Ultra-15 centrifugal filter unit with a 10 kDa molecular-weight cutoff. The protein was further purified using anion-exchange chromatography (Q Sepharose Fast Flow, GE Healthcare). After the protein had been loaded onto the column, it was first washed with 20 m*M* HEPES pH 8.0, 10 m*M* NaCl buffer. The protein was then eluted by a linear gradient to 500 m*M* NaCl in the same buffer. The fractions containing protein were pooled and concentrated for further purification using size-exclusion chromatography (Superdex 75, GE Healthcare) with equilibration in 20 m*M* HEPES pH 8.0, 10 m*M* NaCl. The results of the size-exclusion chromatography clearly suggested that all of the protein eluted as monomers. The fractions containing protein were pooled and used for crystallization and ITC.

### 2.2. Crystallization and structure determination   

#### 2.2.1. Pm-SiaP with Neu5Ac   

Pm-SiaP was concentrated to 44 mg ml^−1^. Additional Neu5Ac was added to the protein at a 1:10 concentration ratio. Trays were set up at this concentration and crystals were grown by the hanging-drop vapour-diffusion method at 4°C using a Mosquito liquid-handling unit (a nanolitre dispensing robot; TTP Labtech). Drops were set up with equal volumes of protein and 1.6 *M* sodium citrate tribasic dihydrate pH 6.5 (crystallization buffer) and were suspended over 100 µl crystallization buffer. SiaP crystals were flash-cooled in mother liquor containing 5%(*v*/*v*) glycerol. The crystals were mounted in loops and X-ray diffraction data were collected on the IMCA-CAT 17-ID beamline at the Advanced Photon Source, Argonne, Ilinois, USA. The X-ray data were processed and scaled using *XDS* (Kabsch, 2010 ▶) and *SCALA* (Evans, 2011 ▶; Winn *et al.*, 2011 ▶).

#### 2.2.2. Fn-SiaP with Neu5Ac   

Crystal trays were set up at a concentration of 58 mg ml^−1^. Additional Neu5Ac was added to the protein at a 1:10 concentration ratio and crystals were grown by the hanging-drop vapour-diffusion method at 4°C using a Mosquito liquid-handling unit (TTP Labtech). Drops were set up with equal volumes of protein and 0.2 *M* MgCl_2_, 0.1 *M* Tris pH 8.5, 35% PEG 4000 (crystallization buffer) and were suspended over 100 µl crystallization buffer. SiaP crystals were flash-cooled in mother liquor containing 5%(*v*/*v*) glycerol. The crystals were mounted in loops and X-ray diffraction data were collected on the PXIII beamline of the Swiss Light Source, Paul Scherrer Institute, Villigen, Switzerland. The X-ray data were processed and scaled using *XDS* (Kabsch, 2010 ▶) and *SCALA* (Winn *et al.*, 2011 ▶).

#### 2.2.3. Vc-SiaP   

Crystallization of the Vc-SiaP protein was carried out in 12-well cell-culture plates (Corning) equilibrated at 4°C by the hanging-drop vapour-diffusion method with a drop consisting of a mixture of equal volumes of protein solution (36 mg ml^−1^ Vc-SiaP in 20 m*M* HEPES, 10 m*M* sodium chloride pH 8.0) and reservoir solution [0.1 *M* MES, 20%(*w*/*v*) PEG 6000 pH 6.0]. The crystals were flash-cooled in mother liquor containing 10%(*v*/*v*) glycerol. The crystals were mounted in loops for data collection at 100 K on the GM/CA-CAT beamline at the Advanced Photon Source, Argonne, Illinois, USA. The data were collected on a MAR CCD detector and were processed and scaled using d*TREK (Pflugrath, 1999 ▶).

### 2.3. Refinement of all protein structures   

For all three structures (Pm-SiaP, Fn-SiaP and Vc-SiaP), molecular replacement was carried out using *Phaser* with the *H. influenzae* Neu5Ac-bound structure (PDB entry 3b50; Johnston *et al.*, 2008 ▶) as the starting model. The starting model was generated from the PDB file (PDB entry 3b50) using *CHAINSAW* in the *CCP*4 package. This program retains the side chains of conserved residues but removes the atoms to the most conserved side-chain atom in the changed residues. All water molecules and ligands were removed. Refinement of the structures was performed using either *PHENIX* (Adams *et al.*, 2011 ▶; for Pm-SiaP and Fn-SiaP) or *REFMAC*5 from the *CCP*4 package (for Vc-SiaP) and model building was carried out using *Coot* (Emsley *et al.*, 2010 ▶). There is only one molecule in the asymmetric unit in all of the crystal forms. The two domains of the protein are tightly held together by the bound ligand in the structures of Fn-SiaP and Pm-SiaP. Hence, refinement of the domains by TLS did not reduce the *R* factor and *R*_free_ in a statistically significant manner. However, we had expected that this would not be the case in the Vc-SiaP structure, as with no ligand bound there is a larger probability of observing domain motion-related effects. However, this was not the case and the primary reason for the lack of this could be the stabilization of the domains by intermolecular contacts mediated by the His tag and cobalt ion. Crystallographic data and refinement statistics are shown in Table 1 ▶. The structure and coordinates have been deposited in the PDB. The PDB code for Pm-SiaP is 4mmp, that for Fn-SiaP is 4mnp and that for Vc-SiaP is 4mag.

### 2.4. Isothermal titration calorimetry (ITC)   

ITC measurements were performed using a MicroCal ITC system (GE Healthcare). The reaction buffer for all of the experiments was 20 m*M* HEPES pH 8.0, 10 m*M* NaCl. All titrations were carried out at 25°C. The protein concentration was determined using the Bio-Rad Bradford reagent. The concentrations of SiaP and Neu5Ac/Neu5Gc were varied in the different titrations and each binding experiment was repeated three times. The nonspecific heat released by the dilution of both the sugars and the protein was calculated by averaging the heat liberated during the last 3–5 injections after saturation. This value was then subtracted from the raw heat released during each injection. To derive the stoichiometric values, the data sets were analyzed using a single-site binding model from the *Origin* ITC analysis software (MicroCal). In this analysis, the values for the stoichiometry, the change in enthalpy (Δ*H*) and the affinity were fitted using nonlinear least-squares analysis. To derive the values for the affinity, Δ*S* and Δ*H*, data sets were globally analyzed using the single-site binding model in *SEDPHAT* (Vistica *et al.*, 2004 ▶; Dam & Schuck, 2005 ▶). The binding affinities of the different proteins and sugars are listed in Tables 2 ▶ and 3 ▶.

### 2.5. Growth-curve experiment: *V. cholerae*   

*V. cholerae* O1 biovar eltor strain N16961 was initially grown on LB agar plates. Subsequently, 3 ml M9 minimal medium supplemented with different sugars (5 mg ml^−1^) was inoculated with bacteria at an OD_600_ of 0.07. These cultures were incubated in a 37°C shaker at 225 rev min^−1^. Optical densities at *A*_600_ were measured at various time points using a Spectronic GENESYS 20 Visible Spectrophotometer to observe the growth pattern of *V. cholerae* in different compounds. The different sugars used were Neu5Ac, Neu5Gc, fetuin (a glycoprotein of foetal serum that contains different sugars and sialic acid; Spiro, 1960 ▶), asialofetuin, galactose, glycerol, d-lactic acid, succinate, sucrose, dl-malic acid and fructose, all at 5 m*M* concentration. Based on the readings, a graph of OD_600_ was plotted.

### 2.6. Enzyme-linked immunosorbent assay (ELISA): *V. cholerae*   

*V. cholerae* was grown on sBHI agar plates containing 100 µ*M* Neu5Ac and LB. Cells were harvested and resuspended in PBS (20 m*M* Na_2_HPO_4_, 150 m*M* NaCl pH 7.0) at an OD_600_ of 0.2. The wells of Costar 96-well EIA/RIA plates (Corning) were filled with 100 µl of the bacterial suspension and six wells were used for each experiment. The plates were dried at 40°C overnight. The plates were washed four times with buffer *C* (72 m*M* sodium acetate, 165 m*M* sodium chloride, 0.55% Tween 20) using an ELx50 Strip Washer (BioTek Instruments, Winooski, Vermont, USA). A neuraminidase digest was performed on three wells by adding 100 µl 0.01 U ml^−1^ neuraminidase (Roche Diagnostics GmbH, Mannheim, Germany) diluted in neuraminidase buffer (50 m*M* sodium acetate, 154 m*M* NaCl, 9 m*M* CaCl_2_, 25 µg ml^−1^ human serum albumin pH 5.6) and the other three wells were treated with just the buffer. The plate was incubated for 6 h at 37°C and the wells were then washed. The monoclonal antibody 3F11, diluted 1/200 in buffer *C*, was added to each well. The plates were incubated at 37°C overnight (approximately 18 h) followed by a wash. The secondary antibody, phosphatase-labelled goat anti-mouse IgM (Kirkegaard and Perry Laboratories, Gaithersburg, Maryland, USA), diluted 1/2000 in buffer *C*, was added. The plates were incubated for 1 h at room temperature and washed. *p*-Nitrophenyl phosphate (1.0 mg ml^−1^; Sigma, St Louis, Missouri, USA) in 8.2% diethanolamine, 0.5 m*M* MgCl_2_ pH 9.8 was added. When a yellow colour had developed, the absorbance at 405 nm was read using a Microplate Autoreader EL311 (BioTek Instruments).

## 3. Results   

### 3.1. Characterization of Fn-SiaP, Pm-SiaP and Vc-SiaP compared with Hi-SiaP   

**Figure 1:**
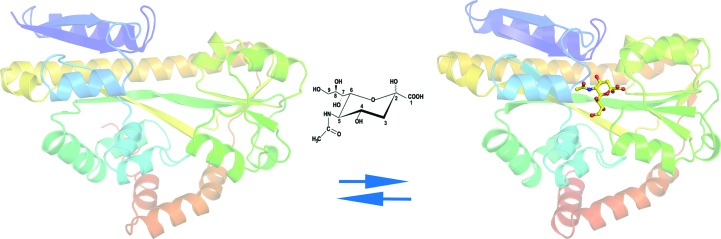
A cartoon diagram showing the unliganded form of Vc-SiaP (in the absence of Neu5Ac) and the ligand-bound form of Fn-SiaP in the presence of Neu5Ac. All structural figures were produced using *PyMOL* (Schrödinger). The structures are coloured as a gradient from blue to red, with the N-­terminus in blue. The figure clearly shows the closure of the domains around the bound ligand (Neu5Ac), which is shown in ball-and-stick representation.

Pm-SiaP and Fn-SiaP were crystallized together with Neu5Ac. Their structures were determined to 1.57 and 2.5 Å resolution, respectively. The structure of Pm-SiaP was refined to an *R* factor of 0.17 and a free *R* factor of 0.20, and that of Fn-SiaP was refined to an *R* factor of 0.21 and a free *R* factor of 0.25. Superposition of the structures of the Neu5Ac-bound forms of the proteins resulted in an r.m.s. deviation of the 301 C^α^ atoms of between 0.4 and 0.6 Å. All three proteins (Pm-SiaP, Fn-SiaP and Hi-SiaP) have very similar structures, distinguished by two domains on either side of a well defined pocket, within which Neu5Ac binds (Fig. 1 ▶). Both domains are made up of residues from both the N-terminal and the C-terminal parts of the sequence. Importantly, two long β-strands and one long α-helix span almost the entire two domains of the protein. The detailed description of the fold is the same as that described earlier for Hi-SiaP.

Vc-SiaP was crystallized without Neu5Ac and we determined its structure to 1.45 Å resolution. These crystals belonged to space group *I*222. The structure was refined to an *R* factor of 0.18 and a free *R* factor of 0.22. However, the r.m.s. deviation between the open form of the Vc-SiaP structure and the open form of the Hi-SiaP structure was about 0.9 Å. Interestingly, a Co atom mediates the inter-protein contact in the crystal lattice. Given that there was no cobalt in our buffers, we propose that this came from the TALON column used in purification. This cobalt appeared to be ligated by three His residues from the C-terminal end of the six-His tag and Glu261 from a neighbouring molecule. It is quite possible that this interaction creates conformational restraint (Supplementary Figs. S1 and S2^1^). The crystallographic data and refinement statistics for Pm-SiaP, Fn-SiaP and Vc-SiaP are shown in Table 1 ▶.

### 3.2. Detailed characterization of the Neu5Ac binding site, hinge regions and their possible interactions   

**Figure 2:**
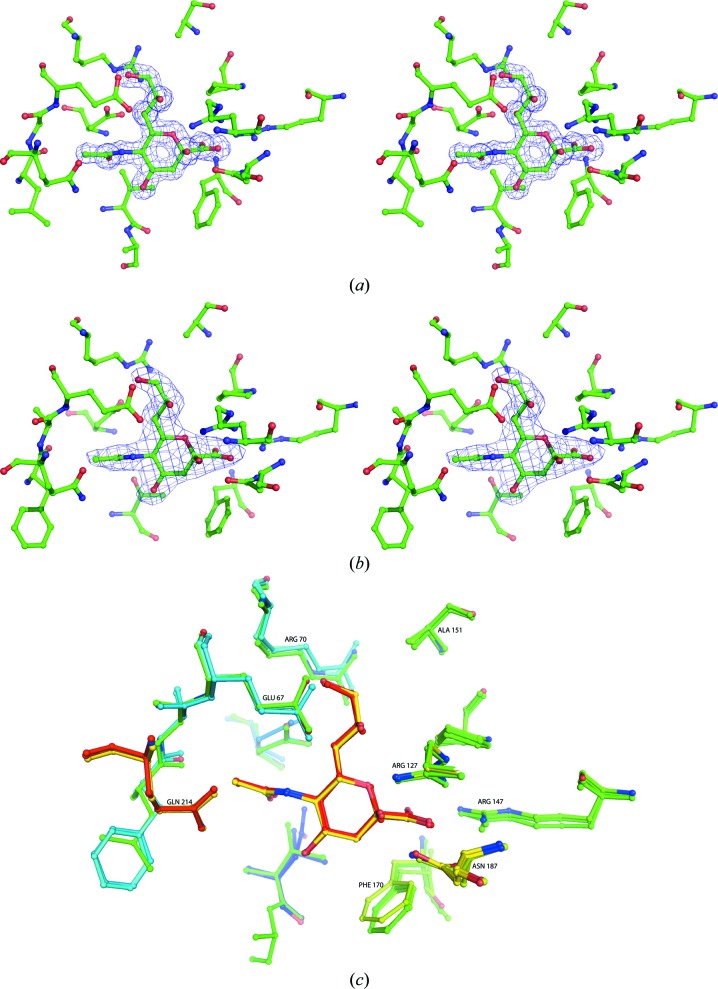
(*a*) Cross-eyed stereo diagram of the bound Neu5Ac and the residues around it in the structure of Pm-SiaP. The electron-density map is a 2*F*_o_ − *F*_c_ map at 1.5 times the r.m.s. deviation. The map was calculated at the end of the refinement. (*b*) Cross-eyed stereo diagram of the bound Neu5Ac and the residues around it in the structure of Fn-SiaP. The electron-density map is a 2*F*_o_ − *F*_c_ map at 1.5 times the r.m.s. deviation. The map was calculated at the end of the refinement. (*c*) A stick diagram showing Neu5Ac and the conserved interacting residues of Hi-SiaP (magenta), Pm-SiaP (blue) and Fn-Siap (cyan). The numbering corresponds to Hi-SiaP.

In all of the Neu5Ac-bound structures, a consistent pattern was seen in how Neu5Ac interacts with four of the residues within the pocket (Fig. 2 ▶). Arg147 interacts with the carboxylate group at C1 of Neu5Ac; this inter­action is conserved among the family of dicarboxylate-binding proteins (Fischer *et al.*, 2010 ▶). Using Fn-SiaP as an example, Arg126 interacts with one of the O atoms of the carboxylate group at the C1 position and the O2 of Neu5Ac to form a second salt bridge. Asn186 interacts with O2 of Neu5Ac, and Asp50 interacts with O7. Similar inter­actions between these four residues and Neu5Ac exist in Hi-SiaP and Pm-SiaP, with minor differences in sequence numbering (*e.g.* Arg127 and Arg147 are involved in the first two interactions in Hi-SiaP), as also seen previously (Müller *et al.*, 2006 ▶; Johnston *et al.*, 2008 ▶). There was one interaction seen in Hi-SiaP (between Asn10 and O10 of Neu5Ac) that was not evident in Fn-SiaP and Pm-SiaP.

**Figure 3:**
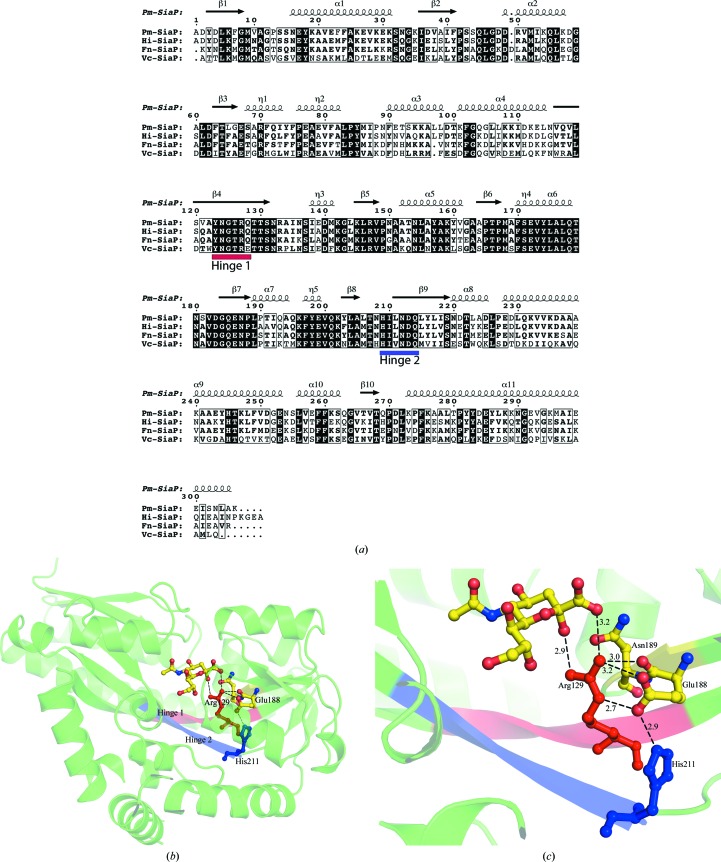
(*a*) Sequence alignment of sialic acid-binding proteins from four different pathogenic bacteria using *ClustalW*2 (EMBL–EBI) and *ESPript*. The hinge regions are marked and labelled. Hinge 1 in shown in red and hinge 2 in blue. (*b*) Structural view of the conserved hinge regions of Fn-SiaP, looking down from the Neu5Ac. Hinge 1 and hinge 2 are coloured red and blue, respectively. Arg129 and His211 are also marked with the corresponding hinge colours. (*c*) Close-up view of conserved residues from the hinge region of Pm-SiaP in the presence of Neu5Ac. The dotted lines show residues interacting by hydrogen/ionic bonds and the distances are labelled in Å.

Previous studies have shown that upon binding Neu5Ac2en Hi-SiaP folds around the ligand, bending primarily at a hinge region that is close to the peptide bonds connecting residues 127–128, 211–212 and 254–255; additional bending was reported close to residues 186–197 (Müller *et al.*, 2006 ▶; Johnston *et al.*, 2008 ▶). In the current study, by superimposing the characterized structures and aligning their corresponding primary sequences, we identified two broader conserved hinge regions that contribute to the opening and closing of the structures. We will refer to them as hinge 1 (YNGTRQ; residues 123–128 in Hi-SiaP) and hinge 2 (HILND; residues 209–213 in Hi-SiaP; Figs. 3 ▶ *a* and 3 ▶ *b*). The overall rotation as described earlier for Hi-SiaP was 28° for Fn-SiaP and Pm-SiaP.

In order to understand the causes that could trigger this rotation upon Neu5Ac binding, we examined those residues common to both the binding site and the connections to the hinge regions in more detail (Fig. 3 ▶ *c*). Interatomic distances between the ligand and charged amino-acid side-chain residues from the hinge region (in the binding pocket) were calculated and we looked for differences in the distances between the domains in the ligand-bound and unbound forms (Tables 4 ▶ *a* and 4 ▶ *b*).

In bound Fn-SiaP, Pm-SiaP and Hi-SiaP (and using the numbering scheme of Fn-SiaP), Arg126 from hinge 1 binds to the O1 and O2 atoms of the bound Neu5Ac (Fig. 3 ▶). Arg126 is part of hinge 1. For these proteins, distances were calculated between Arg126 and its neighbouring atoms in Asn186, Asn211, Glu185, Asn153, crystallographically determined waters and Neu5Ac. Next, for the same bound proteins, the distances between Glu185 and His208 were also calculated. His208 is at the beginning of a long β-strand (hinge 2). Examination of the superimposed structures reveals that these residues (Arg126 and His208) could be the hinges and could operate by changing the hydrogen-bond structure through making and breaking a series of hyrdrogen bonds upon Neu5Ac binding. Again, for the bound proteins, distances were also calculated from Asn211 to water and to Glu67 (Table 4 ▶ *b*).

For unbound forms of Vc-SiaP and Hi-SiaP, a range of interatomic distances involving the same three conserved residues mentioned above were measured (Table 4 ▶ *a*). In the case of Hi-SiaP, a comparison of interatomic distances between Arg127, Glu186, His209 and Neu5Ac in the bound and unbound forms (Tables 4 ▶ *a* and 4 ▶ *b*) shows that in the unbound form Arg127 is closer to Glu186: the distances between OE1 and O and the guanidino-group donor N atoms are 3.1 and 2.6 Å, respectively. In the bound conformation the distances between OE1 and O and the guanidino-group donor N atoms are 3.3 and 2.8 Å, respectively. Thus, in the presence of Neu5Ac Arg127 is closer to Neu5Ac and forms a salt bridge.

### 3.3. Thermodynamic characterization of the binding of Fn-SiaP, Pm-SiaP and Vc-SiaP to Neu5Ac, Neu5Gc and Neu5Ac2en   

**Figure 4:**
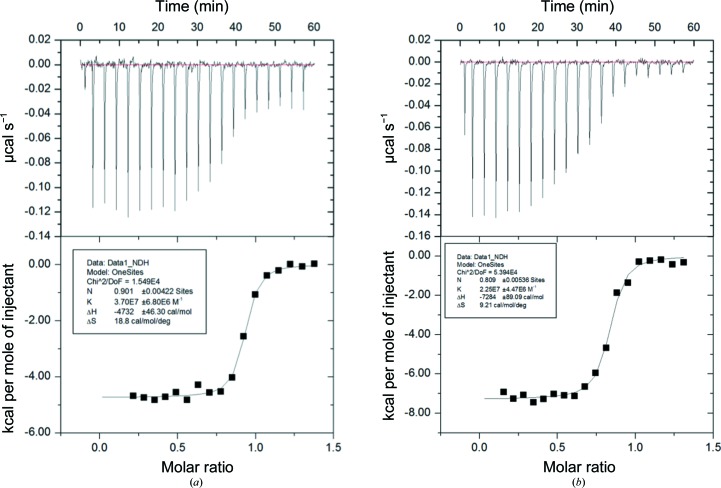
Calorimetric titration isotherms of Pm-SiaP binding to Neu5Ac (*a*) and Neu5Gc (*b*). The top half show the heat change after every injection and the bottom part shows the fit of the curve. Molar ratio is the stoichiometry of the observed binding between the protein and the ligand.

Isothermal titration calorimetry (ITC) was used to measure the binding affinity of SiaPs for Neu5Ac, Neu5Gc and the Neu5Ac analogue *N*-acetyl-2,3-dehydro-2-deoxyneuraminic acid (Neu5Ac2en; Sigma–Aldrich). The isotherms were best fitted using single-site binding, which indicates that these SiaPs bind to Neu5Ac/Neu5Gc in a 1:1 stoichiometric ratio (Fig. 4 ▶). These results suggest that the driving force of binding is enthalpic. There was no measurable interaction with Neu5Ac2en. The binding affinities of Fn-SiaP and Pm-SiaP to Neu5Ac are 45.5 and 19.7 n*M*, respectively. These binding affinities are very similar to that of Hi-SiaP (28 n*M*; Johnston *et al.*, 2008 ▶). Interestingly, the binding affinity of Vc-SiaP is 309 n*M*, *i.e.* ten times weaker than those of Fn-SiaP and Pm-SiaP. We suspected that the value of Vc-SiaP is affected by the Co atom that is bound to the C-terminus (Supplementary Figs. S1 and S2). We then expressed Vc-SiaP in the absence of the six-His tag, purified the protein and carried out the binding studies again. This did not make a difference, suggesting that the His tag plays no role in the affinity (data not shown). The structural features that might contribute to the low binding affinity of Vc-SiaP remain unclear.

### 3.4. Structure–activity relationship studies of Hi-SiaP and its individual residues in the sialic acid-binding pocket using ITC   

In order to determine the role of different amino acids and the conserved interactions in the binding pocket of Hi-SiaP, Johnston and coworkers carried out uptake assays of Neu5Ac by generating mutations of residues in the binding pocket. In order to understand the effect of some of these mutations on the structure and thermodynamics of the protein, we used ITC to measure the binding affinity of eight mutants of Hi-SiaP for Neu5Ac (Table 3 ▶). The isotherms were best fitted with a single-site binding mode, which indicates that the SiaPs bound to Neu5Ac in a 1:1 stoichiometric ratio. The data show that the E67A, E186Q and T64K mutants bind to Neu5Ac with micromolar affinity, while the wild type binds with nanomolar affinity. There was no measurable binding of the T64R mutant to Neu5Ac. These results agree with the respective partial or complete lack of complementation shown by these mutants in whole-cell ELISA tests that were used to quantify the sialyl­ation of lipooligosaccharide (LOS; Johnston *et al.*, 2008 ▶). In contrast, while the binding affinity of N187Q to Neu5Ac was 1.43 µ*M*, it was previously found to show complete complementation. This mutation might have altered some other interactions within the binding pocket. As expected from the previous complementation study, the R127K and the T64R mutants did not bind to Neu5Ac with measurable affinity. We hypothesize a role for His209 as a key residue that is involved in the network of interactions that cause conformational change on sugar binding. If this is the case then the mutation of this His209 to Ala should have a significant effect on the binding affinity. The H209A mutant showed a significantly reduced binding affinity of 662 n*M*.

### 3.5. Growth of *V. cholerae* in the presence of different compounds   

It has been shown previously that strains of *V. cholerae* can use sialic acid alone as a carbon source (Almagro-Moreno & Boyd, 2009 ▶). This finding was supported by our data, which show that *V. cholerae* grows in the presence of Neu5Ac, fetuin alone and fetuin with neuraminidase. Little growth is observed in the presence of succinate, sucrose, dl-malic acid or fructose. No growth is observed in the presence of asialofetunin, Neu5Gc, Neu5Gc with glycerol, galactose, glycerol or d-lactic acid (Supplementary Fig. S3).

### 3.6. Measurement of Neu5Ac incorporation onto surface LOS of *V. cholerae* using ELISA   

3F11 is a monoclonal antibody whose binding to the non­reducing terminal sugars of the bacterial surface lipooligo­saccharides (LOS) is blocked if these LOS are sialylated. Our ELISA tests showed that *V. cholerae* grown with Neu5Ac did not bind 3F11, but that binding did occur after treatment with neuraminidase, which cleaves sialic acid from the LOS (Supplementary Fig. S4). This indicates that *V. cholerae* can incorporate sialic acid into its surface LOS. As has been shown previously, Neu5Ac is involved in *V. cholerae* infections in the gut (Almagro-Moreno & Boyd, 2009 ▶). It is interesting that our experiments also suggest that *V. cholerae* cannot use Neu5Gc as a carbon source.

## 4. Discussion   

All four of the bacterial species that have so far been identified as employing TRAP transporters to help decorate their surfaces with SA are important human or animal pathogens, and for most of them SA has been shown to be important for virulence. In terms of potential therapeutic intervention, the periplasmic solute receptor of the SA-TRAP transporter (SiaP), being present in the accessible perisplasmic space, is an obvious target, and its binding site for SA is of particular interest. Since the SiaP of only one of these species has been characterized (*H. influenzae*), in this study we resolved the structures of the SiaP analogues of all three unstudied species (*P. multocida*, *F. nucleatum* and *V. cholerae*), with special focus on the protein–ligand binding site. As discussed in detail below, our results reveal a remarkable consistency of structure amongst all four species at the level of overall protein structure and in the details of the binding site. Cross-comparison of the ligand-bound SiaP structures has also helped to reveal important new features of arguably the most critical part of the binding site; that is, where the ligand initially establishes contact with the protein, thus setting in motion the folding of the two domains of SiaP around SA.

### 4.1. Similarities of the structures of Hi-SiaP, Fn-SiaP, Pm-SiaP and Vc-SiaP   

The structures of the SiaPs from all four species, when superimposed, showed remarkable congruency considering their disjunct phylogenetic origins (two phyla and three orders). The commonalities of the structures reported here, with a ligand-binding pocket in between two domains, provide further support for the idea that the periplasmic solute receptors of all TRAP transporters have similar structures, despite the fact that (i) they capture a wide variety of ligands and (ii) the average sequence identity between the seven proteins that had been described up to 2010 was only around 20% (Fischer *et al.*, 2010 ▶). The sequence identities of the four proteins, using Hi-SiaP as the reference, are Pm-SiaP, 73%; Fn-SiaP, 65%; Vc-SiaP, 47%.

It is likely that specificity of binding is provided at the binding site. In comparing the sequences of eight SA SiaPs, Müller *et al.* (2006 ▶) noted many conserved regions, and several of these have been the focus of structural studies of the binding site and proposed hinge regions of Hi-SiaP. The process, in a general sense, is thought to involve two components: firstly the establishment of links between the ligand and the protein, followed by the establishment of new links within Hi-SiaP itself, which also requires the rearrangement of some existing protein–protein links. The two previous structural studies of Hi-SiaP (Müller *et al.*, 2006 ▶; Johnston *et al.*, 2008 ▶) particularly the study of Johnston and coworkers (which used the high-affinity natural ligand Neu5Ac), have indicated that it is the atoms at the C1 end of Neu5Ac that are probably the first to bind to Arg147 of Hi-SiaP. The study of Johnston and coworkers gave particular importance to Arg127, whose δ-guanido group interacts with both the C1 carboxyl and the C2 hydroxyl of Neu5Ac in forming a salt bridge. The crucial importance of Arg127 in the binding process was further supported in their study by the complete loss of complementation in an ELISA test of a mutant of this residue (R127A) and in our study by the lowered binding affinity of R127A and R127K (a more conservative mutation) for Neu5Ac. One of the other residues investigated in the study of Johnson and coworkers was His209. The mutants H209A and H209Q also showed complete complementation. As seen in our binding data, the mutation at this position only decreased the binding affinity but did not abolish it. If His209 is involved in the opening and closing of the domains, it does this by stabilizing the closed conformation when the ligand is bound and the open conformation otherwise. Mutation of this residue will affect the kinetics of opening and closing rather than of binding itself, which could explain why full complementation was observed in spite of a reduced binding affinity.

### 4.2. A central role for His209 in ligand binding and protein folding   

Our cross-comparison of the four SiaP structures allows us to propose a crucial role for Arg127 and His209, which are at the centre of a conserved hydrogen-bonding network, in the establishment of both the initial ligand–protein link and the new protein–protein links that initiate and stabilize the folding of Hi-SiaP and the other SiaPs. We suggest the following sequence, using Hi-SiaP residue numbering for convenience. In the unbound form, Glu186 forms a tight hydrogen bond (a salt bridge of NE and NH2 with OE1 and OE2) with Arg127 and a poor interaction with His209. When the highly charged Neu5Ac binds to the protein, Arg127 (*via* NH1 and NH2) now forms a salt bridge with one of the carboxylate O atoms (O1B) and the O2 bound to C2. This weakens the interaction between Arg127 and Glu186, which can now form a stronger interaction with His209. The hydrogen-bonding pattern around His209 is now altered, triggering a conformational change of Hi-SiaP that has been previously implicated in the protein-folding process (Müller *et al.*, 2006 ▶). The bond between His209 and Glu186 will also help to stabilize the folded form. The binding of the charged Neu5Ac and the resulting changes in the environment might also change the p*K*_a_ of His209.

### 4.3. Bacterial SiaPs can bind variants of SA that are not typical of their host   

The two main forms of SA found in animals are Neu5Ac and Neu5Gc. Most animals have both forms, but Neu5Gc is not produced by humans and at least some birds (Varki, 1992 ▶, 2009 ▶). Of the four pathogens that we studied here, *H. influenzae*, *V. cholerae* and *F. nucleatum* are human pathogens and *P. multocida* is a pathogen of many animals, including humans and particularly birds. Thus, it was interesting that the SiaPs of *F. nucleatum* and *P. multocida* bound both forms of SA with equal affinity, and that there was only a moderate decrease in the binding of Neu5Gc in the case of *V. cholerae*. This is probably explained by the fact that the chemical difference between Neu5Ac and Neu5Gc does not occur around the C1 region but at the C5 atom of the sugar, which is thus distant from the initial binding site between SA and the SiaP. In other words, the specificity of SA binding, as suggested above, is determined by the atoms at the C1 end of any SA. Interestingly, even *E. coli* has the ability to use Neu5Ac and Neu5Gc as a carbon source, suggesting that the observed phenomenon is more common in bacteria than initially thought (Hopkins *et al.*, 2013 ▶). The same authors also showed that the *E. coli* transport system for these sugars (NanT) can be replaced by the *H. influenzae* SiaPQM system to transport both Neu5Ac and Neu5Gc into *E. coli*. This provides evidence for the fact that it is the same system that transports both Neu5Ac and Neu5Gc and that our studies showing that the binding affinity of all of the proteins to both sugars is physiologically relevant.

### 4.4. *V. cholerae* can incorporate SAs into its surface glycolipids   

While bioinformatics analysis has supported the idea that all of these bacteria have the enzymes to incorporate SA as the terminal compound in surface glycolipids (Jones *et al.*, 2002 ▶), there was no direct evidence that this would in fact happen. Our results show that it can, and suggest that this might also be true for other species that have a conserved SA transport pathway and the enzymes for incorporation. Interestingly, this pathway plays no role in the common infection of the GI tract caused by *V. cholerae*. Currently, very little is known regarding the role of this system in pathogenesis; however, one could speculate that, similar to *H. influenzae*, *V. cholerae* could also be able to avoid immune response in the serum, providing yet another model of *V. cholerae* infection.

## 5. Conclusion   

As well as providing fundamental information about the structure of the sialic acid TRAP transporters of three previously uncharacterized pathogenic species, the findings of this study further our understanding of how SAs bind to TRAP transporters in general. By pinpointing, at the residue level, those regions of the protein most critical to SA binding, and showing that these are common to all known SA TRAP transporters, this study lends weight to the idea that these transporters are promising targets for therapeutic intervention.

## Supplementary Material

Supporting Information.. DOI: 10.1107/S139900471400830X/wa5064sup1.pdf

PDB reference: sialic acid-binding protein from *Pasturella multocida*, 4mmp

PDB reference: from *Fusobacterium nucleatum*, 4mnp

PDB reference: from *Vibrio cholerae*, 4mag

## Figures and Tables

**Table 1:** Data-collection and refinement statistics for SiaP structures Values in parentheses are for the highest resolution shell. All coordinates and data have been submitted to the Protein Data Bank.

	Fn-SiaP	Pm-SiaP	Vc-SiaP
Resolution range ()	41.842.50 (2.652.50)	85.521.57 (1.651.57)	11.731.45 (1.5021.45)
Wavelength	1.00	1.00	0.9793
Space group	*P*2_1_2_1_2_1_	*P*2_1_2_1_2_1_	*I*222
Unit-cell parameters			
*a* ()	41.84	42.64	74.98
*b* ()	58.73	77.76	82.32
*c* ()	111.67	85.52	119.35
= = ()	90	90	90
Total reflections	61560	248102	394752
Unique reflections	10012	40254	65409 (6477)
Multiplicity	6.1 (6.4)	6.2 (5.3)	6.04 (5.97)
Completeness (%)	99.6 (99.9)	99.2 (95.7)	99.7 (100.00)
Mean *I*/(*I*)	13.6 (7.8)	13.1 (2.3)	8.9 (2.0)
Wilson *B* factor (^2^)	26.3	20.3	24.6
*R*_merge_	0.09 (0.19)	0.09 (0.44)	0.07 (0.65)
*R* factor	0.21 (0.26)	0.17 (0.21)	0.18 (0.32)
*R*_free_	0.25 (0.30)	0.20 (0.25)	0.22 (0.34)
No. of atoms			
Total	2573	2611	2790
Macromolecules	2411	2410	2458
Ligands	21	21	6
Water	141	180	320
No. of protein residues	301	309	307
R.m.s.d., bonds ()	0.002	0.01	0.011
R.m.s.d., angles ()	0.537	1.27	1.24
Ramachandran favoured (%)	99	100	99
Ramachandran outliers (%)	0	0	0
Average *B* factor (^2^)			
Overall	26.18	22.63	29.40
Macromolecules	26.17	22.26	28.00
Neu5Ac	22.52	14.95	
Solvent	25.96	28.50	40.10
PDB code	4mnp	4mmp	4mag

**Table 2:** Binding affinities of different SiaPs to different sugars and entropic and enthalpic components MicroCal ITC was used for the measurements and the data were analyzed using the *Origin* software.

SiaP	Ligand	*K*_d_ (n*M*)	*H* (calmol^1^)	*T* *S* (calmol^1^)
*F. nucleatum*	Neu5Ac	45.5	9165	849.3
	Neu5Gc	45.7	12074	2059.4
*P. multocida*	Neu5Ac	19.7	6560	3948.2
	Neu5Gc	30.7	7469	2777.3
*V. cholerae*	Neu5Ac	306	7175	508.9
	Neu5Gc	1090	5200	1127.6
*H. influenzae* †	Neu5Ac	28	15300	5000

† Thermodynamic data for Hi-SiaP with Neu5Ac are taken from Johnston *et al.* (2008 ▶) for comparison with other SiaPs.

**Table 3:** Binding affinities of wild-type Hi-SiaP and different mutants of Hi-SiaP to Neu5Ac and entropic and enthalpic components MicroCal ITC was used for the measurements and the data were analyzed using the *Origin* software.

Hi-SiaP (with Neu5Ac)	*K*_d_	*H* (calmol^1^)	*T* *S* (calmol^1^)
Wild type	16.6n*M*	17500	6883
E67A	19.8*M*	249.5	6168.6
E186Q	1.78*M*	6827	1016
N187Q	1.41*M*	7939	40.826
T64K	0.641*M*	8743	294.72
H209A	0.662*M*	13360	4917
T64R	No measurable binding		
R127K	No measurable binding		

**(a):** Interatomic distances between the conserved residues from the hinge region of Vc-SiaP and Hi-SiaP. Residue numbers correspond to Hi-SiaP. All distances are in .

Residue	Atom	Residue	Atom	Vc-SiaP	Hi-SiaP
Arg127	NH1	Asn187	ND2	3.3	3.4
	NH1		WAT	2.9	2.4
	NH2	Asn187	ND2	3.1	3.3
	NH2	Glu186	OE1	3.1	3.1
	NH2	Glu186	OE2	3.5	3.4
	NH2	Asn154	OD1	3.7	3.4
	NE	Glu186	OE2	2.7	2.6
	NE	Asn154	OD1	3.4	3.3
Glu186	OE2	His209	NE2	2.7	2.6
Asn212	OD1		WAT	2.8	3.3
	ND2		WAT	2.8	3.2
	ND2	Glu67	OE1	3.9	3.0

**(b):** Interatomic distances (in ) between the conserved residues from the hinge region of Hi-SiaP, Pm-SiaP and Fn-SiaP in the presence of Neu5Ac. Residue numbers correspond to Fn-SiaP.

Residue	Atom	Residue	Atom	Fn-SiaP	Pm-SiaP	Hi-SiaP
Arg126	NH1	Asn186	ND2	3.5	3.4	3.5
	NH1		WAT	3.3	3.3	3.2
	NH1	Neu5Ac	O2	3.0	2.9	2.9
	NH1	Asn211	OD1	4.1	4.1	4.0
	NH2	Asn186	ND2	3.4	3.4	3.4
	NH2	Glu185	OE1	3.5	3.8	3.9
	NH2	Neu5Ac	O2	3.4	3.3	3.2
	NH2	Neu5Ac	O1A	3.3	3.2	3.1
	NE	Glu185	OE1	2.3	2.7	2.8
	NE	Asn153	ND2	3.4	3.4	3.5
Glu185	OE1	His208	NE2	2.9	2.8	2.7
Asn211	OD1		WAT	3.2	3.2	3.2
	ND2		WAT	2.7	2.7	2.8
	ND2	Glu67	OE2	3.8	3.7	3.8

## Footnotes

1 Supporting information has been deposited in the IUCr electronic archive (Reference: WA5064).
