# Supplementary material for: Bacterial periplasmic sialic acid-binding proteins exhibit a conserved binding site
Source: Acta Crystallogr D Biol Crystallogr. 2014 Jun 24;70(Pt 7):1801–11. doi: 10.1107/S139900471400830X (PMC4089482; doi:10.1107/S139900471400830X)
Supplement: Supplementary file 1 [file d-70-01801-sup1.pdf]

Acta Crystallographica Section D

Volume 70 (2014)

Supporting information for article:

Bacterial periplasmic sialic acid binding proteins exhibit a conserved binding site

Thanuja Gangi Setty, Christine Cho, Sowmya Govindappa, Michael A. Apicella and S. Ramaswamy

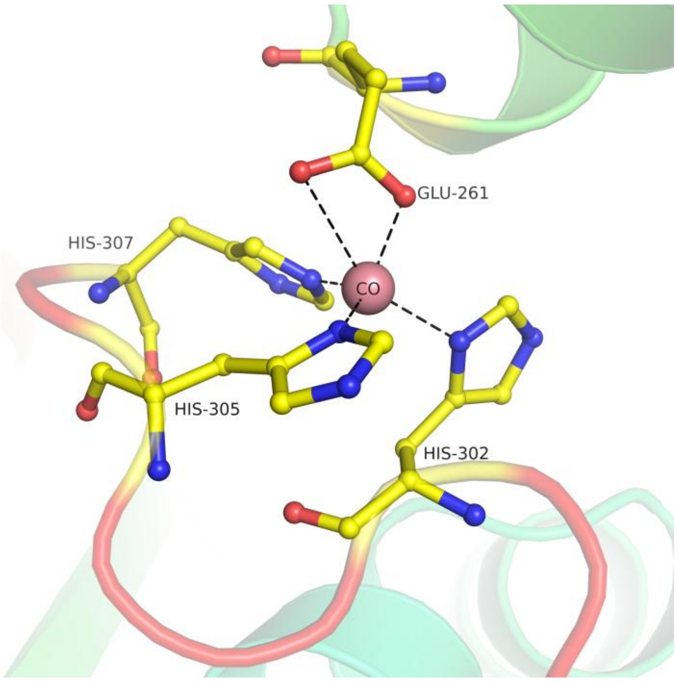

(a)

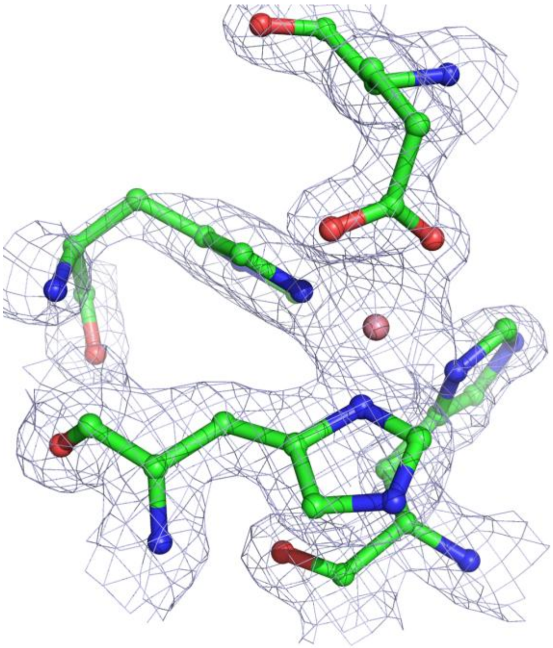

(b)

Figure S1 **(a)**. Close-up view of Vc-SiaP illustrates, cobalt atom showing interaction with three His-residues form C-terminal His-tag and Glu-261 of the neighboring molecule. **(b)**. Electron density map of the cobalt atom and the residues that it is liganded to. The cobalt most probably leached from the metal affinity column used for protein purification. The cobalt atom is shown as a sphere in magenta.

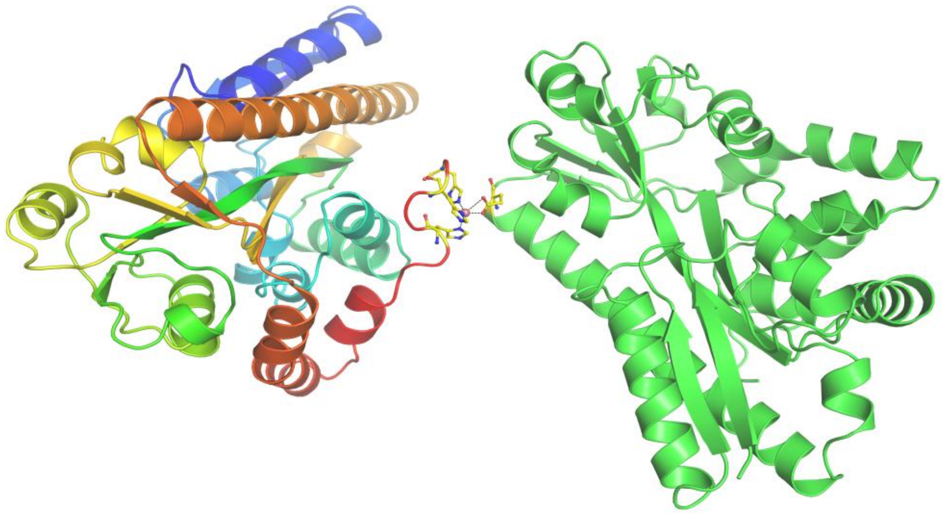

Figure S2 Cobalt atom forming inter-protein contact in the crystal lattice in Vc-SiaP. These interactions spread over the entire crystal along one direction and stabilizes the lattice contacts.

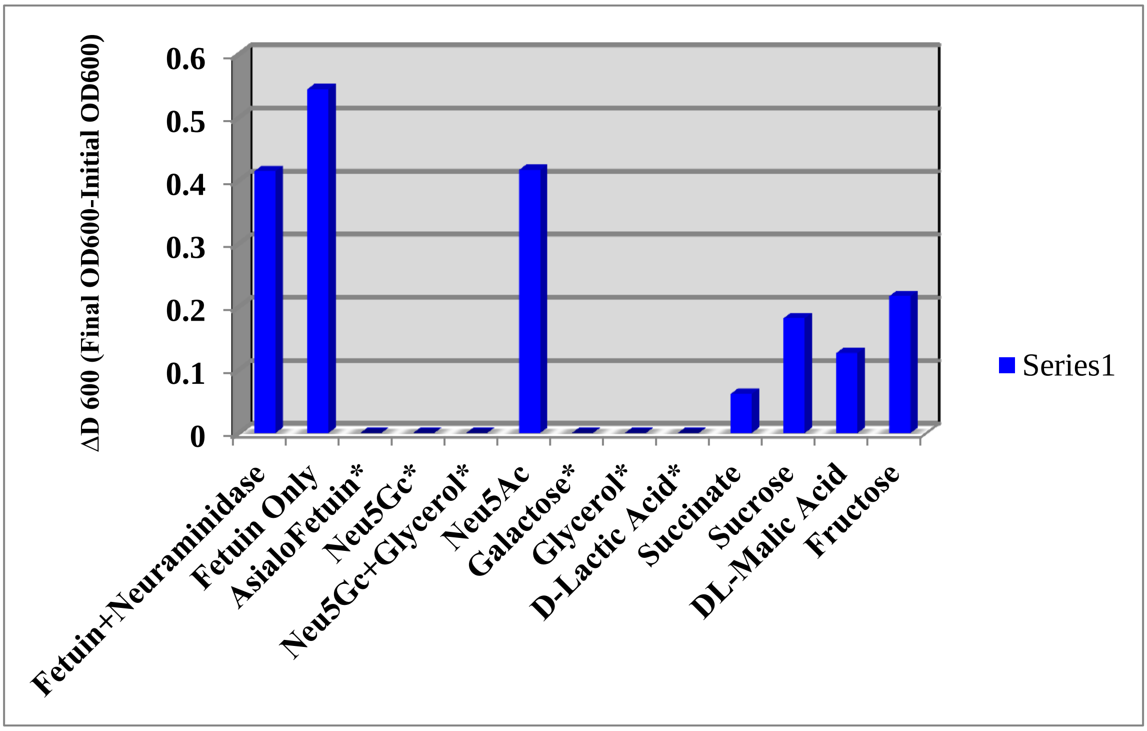

Figure S3 Growth of *V. cholerae* in presence of different sugars. *Vibrio cholerae* O1 biovar eltor str. N16961 was grown in M9 minimal medium supplemented with different sugars were incubated at 37°C and Optical densities at A_{600} were measured at various time points using Spectronic 20 Genesys™ (Spectronic). Clearly growth is observed in Neu5Ac. Surprisingly, no growth is observed when grown in Neu5Gc.

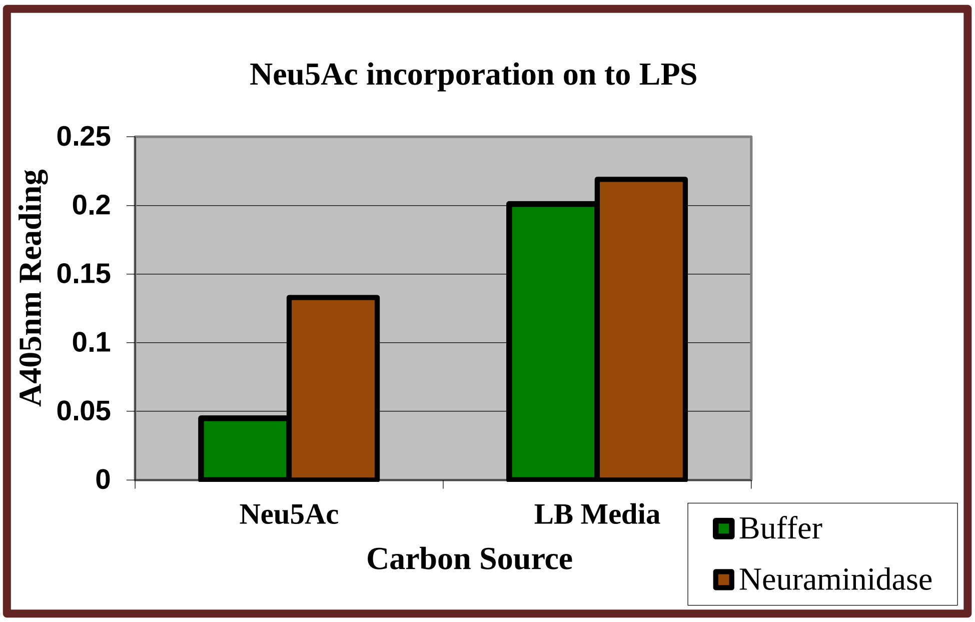

Figure S4 Measurement of Nue5Ac incorporation on to LPS using ELISA based assay. The 3F11 antibody binds to the terminal sugar of the gram negative bacterial LOS and does not bind to Neu5Ac. The difference in the observed absorbance (before and after adding neuraminidase) is a measure of the level of the incorporation of Neu5Ac. As seen there is very little difference in the control (LB media), but a significant difference when grown with Neu5Ac.

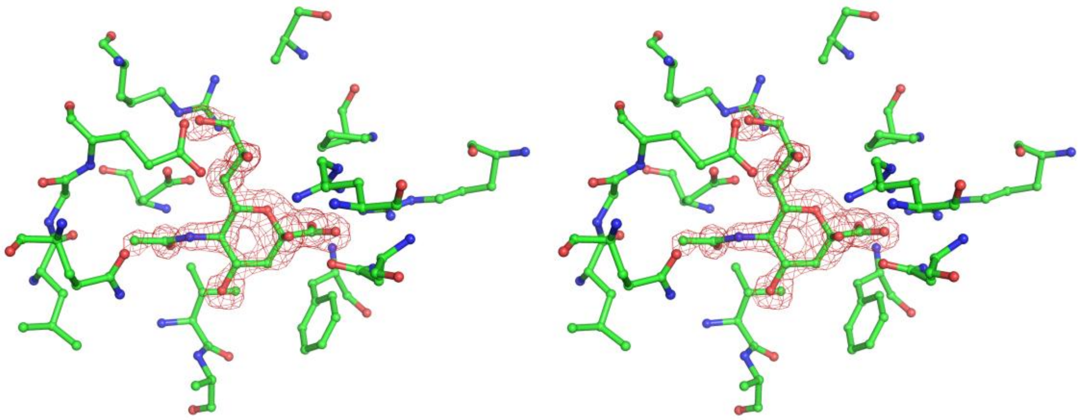

(a)

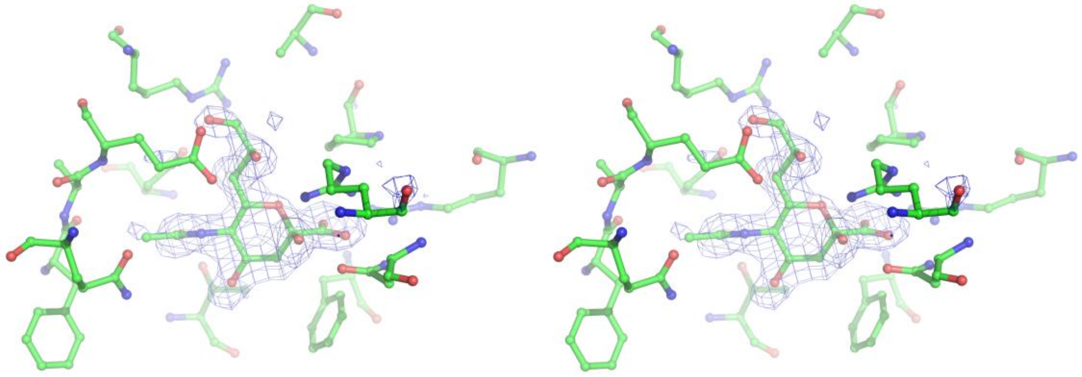

(b)

Figure S5 Stereo diagrams of the difference electron density maps demonstrating the presence of Neu5Ac in the binding pocket. **(a)** Fo-Fc map at 2.0 sigma after molecular replacement showing the presence of Neu5Ac in the active site. The electron density map is superposed on the refined structure of the final refined Pm-SiaP structure. **(b)** Simulated annealed omit map (FO-FC), calculated in Phenix of the Fn-SiaP structure. The Neu5Ac was

omitted. The electron density map is superposed on the refined structure of the final refined Fn-SiaP structure. The map is contoured at 3 times the RMS.
